# Therapeutic Potential of Targeting the Th17/Treg Axis in Autoimmune Disorders

**DOI:** 10.3390/molecules22010134

**Published:** 2017-01-14

**Authors:** Patrizia Fasching, Martin Stradner, Winfried Graninger, Christian Dejaco, Johannes Fessler

**Affiliations:** Department of Rheumatology and Immunology, Medical University of Graz, Auenbruggerplatz 15, 8036 Graz, Austria; patriziafasching@gmx.at (P.F.); martin.stradner@medunigraz.at (M.S.); winfried.graninger@medunigraz.at (W.G.); johannes_fessler@hotmail.com (J.F.)

**Keywords:** Th17-cells, regulatory T-cells, autoimmunity, RORγt, Foxp3

## Abstract

A disruption of the crucial balance between regulatory T-cells (Tregs) and Th17-cells was recently implicated in various autoimmune disorders. Tregs are responsible for the maintenance of self-tolerance, thus inhibiting autoimmunity, whereas pro-inflammatory Th17-cells contribute to the induction and propagation of inflammation. Distortion of the Th17/Treg balance favoring the  pro-inflammatory Th17 side is hence suspected to contribute to exacerbation of autoimmune disorders. This review aims to summarize recent data and advances in targeted therapeutic modification of the Th17/Treg-balance, as well as information on the efficacy of candidate therapeutics with respect to the treatment of autoimmune diseases.

## 1. Introduction

Autoimmunity comprises a number of pathological conditions and disorders with distinct appearances and characteristics, all sharing the common hallmark of impaired self-tolerance. In healthy individuals, the mechanisms of central and peripheral tolerance ensure a proper regulation of the immune system thus preventing autoimmunity. Regulatory T-cells (Tregs) are crucial for the maintenance of peripheral immunological tolerance. Impairments in Treg numbers or function have been investigated in various autoimmune diseases (AIDs) [1]. More recently, autoimmunity has been linked to an impaired balance between Tregs and Th17-cells, an effector T-cell subset described to promote inflammation by acting as Treg antagonists. A shift in the Th17/Treg equilibrium towards the pro-inflammatory Th17 side has been reported in several autoimmune disorders including rheumatoid arthritis (RA), ankylosing spondylitis (AS), psoriasis and psoriatic arthritis (PsA), multiple sclerosis (MS), systemic lupus erythematosus (SLE), inflammatory bowel disease (IBD), as well as Crohn’s disease (CD) [2,3,4]. Moreover, several drugs with the potential to modify Treg- and in particular Th17-responses, have already shown to be effective and received approval for the treatment of some of these disorders [5,6,7,8,9,10,11]. These findings have corroborated existing evidence on the involvement and reciprocal role of Tregs and Th17-cells in autoimmune inflammation, thus highlighting the importance of investigating new therapeutic strategies in this regard. This review aims to summarize recent data and advances in targeted therapeutic modification of the Th17/Treg-balance, as well as on the efficacy of candidate therapeutics with respect to the treatment of AIDs.

## 2. Treg-Cells

Although a wealth of regulatory immune cells [12,13] have been identified, CD4+ Tregs remain the most extensively studied cell-type with immunosuppressive properties. Tregs can use a variety of mechanisms to induce immunosuppression, including indirect suppression via the expression of inhibitory cytokines, metabolic disruption of target T-cells, cytolysis, and regulation of dendritic cell maturation and function [14]. Tregs are divided into two different subsets, depending on their origin. While natural Tregs (nTregs) are derived from the thymus, inducible Tregs (iTregs) develop from naïve T-cell precursors in the periphery. Thymic development of nTregs depends on TCR-stimulation in combination with CD28 co-stimulation. CD28 is furthermore essential for homeostatic proliferation and survival of nTregs in the periphery [15]. In contrast, development of iTregs requires the presence of IL-2 and transforming growth factor (TGF)-β instead of CD28 co-stimulation [16].

### 2.1. Transcription Factors and Surface Markers

Treg-cell lineage commitment is pivotally promoted by the expression of transcription factor forkhead box P3 (Foxp3). Divers mutations in the human *FOXP3* gene result in an allotropic syndrome of multi-organ autoimmunity (immune dysregulation, polyendocrinopathy, enteropathy, X-linked (IPEX) syndrome [17]) and similar effects were observed in the *scurfy* mouse, a murine model carrying a mutation in *Foxp3* [18]. Moreover, continued expression of Foxp3 is required to maintain function and lineage identity of mature peripheral Tregs [19]. Transcription of *FOXP3* is activated by signal transducer and activator of transcription (STAT) 5, another transcription factor shown to influence Treg differentiation and survival [20].

Foxp3 is one of the most specific markers to identify Tregs; however, Foxp3+ cells without suppressive function are also present in humans. Another notable limitation of this marker is the fact, that cells must be permeabilized in order to stain Foxp3 intracellularly. Permeabilized cells are not viable anymore and can thus not be used for further functional testing. Other, reliable markers expressed on the surface of Tregs are needed to identify this cell population for functional experiments.

Initially, Treg characterization was mainly based on their elevated expression of surface IL-2 receptor α-chain (CD25) until it was evident that CD25 can also be elevated in activated T-cells lacking suppressive function. Various additional surface-markers were proposed to define Tregs including cytotoxic T-lymphocyte associated Ag-4 (CTLA-4) [21], adhesion molecule CD62L [22], glucocorticoid-induced tumor necrosis factor receptor (GITR) [23], programmed cell death-1 (PD-1) [24] and many others, while CD49d, CD127, CD26 and CD6 were proposed as negative markers [25,26,27,28,29]. However, as none of these molecules is Treg-specific, the use of a combination of several markers is now recommended for a reliable identification of Tregs.

### 2.2. Cytokines

One of the features attributed to Tregs is the secretion of cytokines exerting suppressive function on various immune cell subsets. The major Treg-cytokines include TGF-β and interleukin (IL)-10 [14]. TGF-β is pivotal for the maintenance of immunological tolerance through interference with differentiation, proliferation and survival of lymphocytes and other immune cells [30]. Targeted deletion of the TGF-βRII receptor in T-cells resulted in early-onset lethal autoimmunity in mice [30,31]. Moreover, T-cell specific TGF-βRII deficiency resulted in the emergence of a highly pathogenic T-cell population overexpressing granzymes, perforin, death receptor ligand FasL and interferon (IFN)-γ, which has been assumed to cause this autoimmune disease [31]. Tregs, however, are not the sole source of TGF-β secretion, and there is a multiplicity of effects exerted by TGF-β on its target cells, as reviewed in detail elsewhere [32,33]. IL-10, in contrast, seems to be predominantly essential for the control of inflammation at environmental interfaces such as lungs and colon. IL-10 does not only induce suppression of pathogenic Th17-cell responses [34,35,36], it is also required to maintain Treg suppressive activity and expression of Foxp3. Besides, IL-10 was reported to interfere with Th1-cell migration to intestinal inflammatory sites [37,38]. Apart from that, nTregs were reported to be a natural source of IL-35, thereby triggering differentiation of naïve T-cells into a distinct iTreg-subset exerting its suppressive function exclusively via production of IL-35 (iTr35-cells) [39]. Notably, iTr35-cells differ from Foxp3+ Treg-subsets as they lack Foxp3 expression [39]. Presumably, IL-35 is required for maximal suppressive function of Foxp3+ Tregs and has been suggested to contribute to the maintenance of immune tolerance in the gut [39,40,41]. The exact physiological role of IL-35 in vivo, however, is under debate and requires further investigation.

## 3. Th17-Cells

Since their identification, Th17-cells have been extensively studied and were soon accepted as a distinct CD4+ helper T-cell lineage [42,43]. A bulk of evidence from numerous studies has demonstrated their malicious, pro-inflammatory involvement in various autoimmune disorders [2]. However, this cell subset also has a physiologic role in the immune system by conferring protective function against microbial pathogens (including fungi, bacteria, and viruses) at mucosal surfaces [44,45].

### 3.1. Transcription Factors and Surface Markers

**Figure 1:**
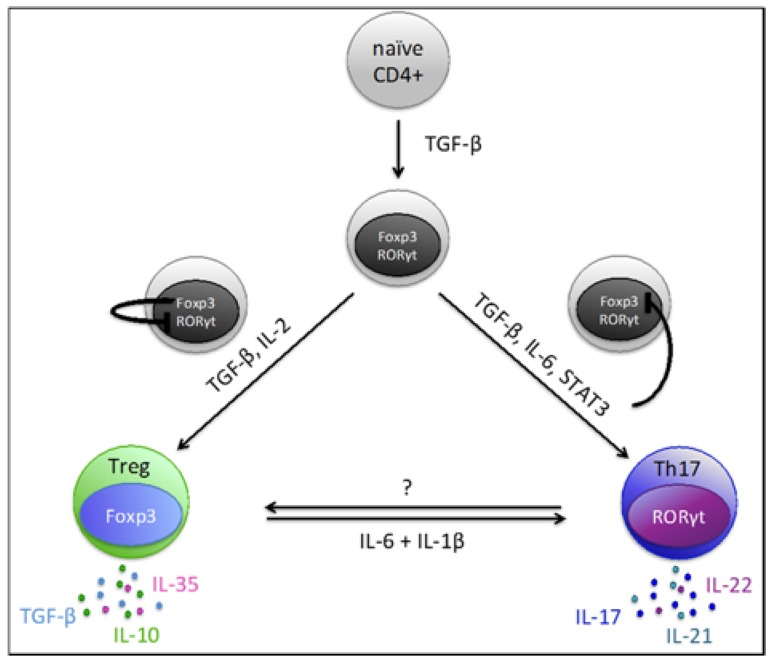
Development and trans-differentiation pathways of Tregs and Th17-cells.

The differentiation of Th17-cells is directed by their master transcription factor retinoic acid-related orphan receptor γt (RORγt), a specific transcript of the *RORC* gene. Interestingly, TGF-β is initially required for the development of both, iTregs and Th17-cells, by triggering the expression of their differentiating transcription factors, Foxp3 and RORγt, respectively [46]. In fact, both transcription factors are initially up-regulated after naïve CD4+ T-cells encounter TGF-β [47]. Whether subsequent differentiation of the cells is skewed towards a regulatory phenotype or a pro-inflammatory Th17 phenotype depends mainly on the surrounding cytokine milieu. Up-regulated Foxp3 initially binds to RORγt thereby inhibiting the development of Th17-cells and favoring Treg development [48]. However, the presence of pro-inflammatory cytokines such as IL-6 and IL-21 can abrogate this TGF-β-dependent, Foxp3-mediated inhibition of RORγt via activation of transcription factor STAT3 [46,49] (Figure 1). Eventually, this leads to the differentiation of Th17-cells and up-regulation of the receptor IL-23R. IL-23 then provides a positive feedback loop for the maintenance, expansion and proper function of Th17-cells [50]. Increased surface expression of IL-23R is characteristic for Th17-cells and might be used to identify this cell population [51]. Further surface markers suggested to serve as Th17-identifiers are chemokine receptors CCR6 and CCR4, both associated with skin homing of cells [52]. Moreover, CXCR3 negativity helps to distinguish CCR6+ Th17-cells from CXCR3+ CCR6+ Th1/17-cells. The latter is a distinct T-cell population exhibiting both Th1 and Th17 characteristics. More recently, the C-type lectin CD161 was reported to be a useful surface marker for Th17-cell characterization given that IL-17 producing cells originate from CD161+ T-cells [53]. A combination of different markers, however, might be the best approach for reliable Th17-cell identification (similar to the strategy to identify Tregs as discussed above).

### 3.2. Cytokines

IL-17A (commonly referred to as IL-17) and IL-17F are the key effector cytokines of Th17-cells. During Th17-cell differentiation, RORγt directly binds to the promoter region of IL-17A, thereby orchestrating its transcription [48]. IL-17 is involved in inflammatory responses by stimulating cell types of both, immune and non-immune nature. The effect of IL-17 in this context is to indirectly promote neutrophil recruitment via induction of CXCL8 in macrophages, epithelial and endothelial cells and fibroblasts [54]. Additionally, tumor necrosis factor (TNF)-α and granulocyte-macrophage colony-stimulating factor (GM-CSF) secreted by Th17-cells also contribute to neutrophil recruitment, activation and survival [55]. Furthermore, IL-17 induces CCL20 expression in various cell types found at sites of chronic inflammation, thereby enhancing attraction of additional Th17-cells, which express CCR6, the receptor for CCL20. Other molecules such as IL-22, IL-26 and IL-21 are also part of the characteristic Th17-signature [47,49,56]. These cytokines feed another positive loop amplifying the Th17 response in an autocrine manner [50,57]. IL-22 seems to be crucially involved in the pathogenesis of psoriasis and RA [58,59] whereas IL-26 most likely contributes to the pathogenesis of intestinal inflammation [60].

### 3.3. Pathogenic vs. Non-Pathogenic

Although Th17-cells were first recognized due to their pathogenic involvement in autoimmune inflammation, accumulating evidence points toward a considerable heterogeneity of the Th17-lineage. For example, a non-pathogenic subset of Th17-cells has been identified, which has a physiologic role in the protection of intestinal barrier function [44,45]. This led to the conclusion that Th17-cells are not uniform in function. Further analyses revealed that Th17-cell differentiation in the presence of TGF-β1 and IL-6 results in cells co-producing IL-17 and IL-10 [61]. These cells are assigned to the non-pathogenic subset of the Th17-lineage, as they are incapable of promoting autoimmune inflammation and might even act anti-inflammatory. On the other hand, differentiation of highly pathogenic Th17-cells from naïve precursor-cells was reported to occur independently of TGF-β signaling in presence of IL-23, IL-6 and IL-1β [62,63]. Lee et al. [64] reported that IL-23 exposure resulted in a transcriptomic switch in differentiating Th17-cells thereby triggering Th17-pathogenicity via induction of a set of unique transcription factors and induction of TGF-β3 production. They support their concept by demonstrating that TGF-β3 and IL-6 were sufficient to induce differentiation of pathogenic Th17-cells in in vitro experiments [64]. More recently, the serum/glucocorticoid regulated kinase 1 (SGK1) was reported to be critical for the generation of pathogenic Th17-cells by deactivating Foxo1, a direct repressor of IL-23R expression [65]. CD5L is another candidate molecule described to regulate Th17-pathogenicity. Loss of CD5L converts non-pathogenic Th17-cells into pathogenic ones that induce autoimmunity [66]. The mechanism by which CD5L regulated Th17-pathogenicity is based on alterations of the cellular fatty acid composition, eventually resulting in RORγt ligand restriction [66]. Collectively, these studies indicate that there exist various subsets of Th17-cells with distinct function and pathogenic capacity.

## 4. Th17/Treg Plasticity and Balance

Shared requirement of TGF-β and the reciprocal regulation of their master transcription factors RORγt and Foxp3 suggest a dichotomy in the generation of Th17-cells and Tregs. More importantly, there is a reported phenotypic and functional plasticity in both populations allowing differentiated cells to “re-”differentiate (Figure 1).

For example, IL-6 does not only enhance Th17-differentiation via STAT3 activation in naïve T-cells but was also shown to promote re-differentiation of Tregs into Th17-cells in presence of TGF-β and IL-1 [67]. Similar observations were published by Deknuydt et al. [68] after combined in vitro treatment of isolated Tregs with IL-6 and IL-1β, but not with IL-6 alone . This finding is in line with the previously reported resistance of TGF-β-induced Tregs to be skewed into the Th17-direction in a cell culture setting with IL-6 and IL-2 [69]. This result highlights the importance of IL-1β in this re-polarization process. A recent study by Komatsu et al. [70] reported CD25^lo^Foxp3+CD4+ T-cells obtained from *Foxp3*^hCD2^ knock-in mice to preferentially loose Foxp3-expression and acquire expression of IL-17 upon adoptive transfer into arthritic mice. These exFoxp3 Th17-cells eventually accumulate in inflamed joints of these animals. Conversely, Gagliani et al. [71] more recently reported reprogramming of inflammatory Th17-cells into IL-10 producing cells with regulatory functions. Clustering analysis of Th17-relevant genes revealed that these exTh17-cells have undergone transcriptional reprogramming and subsequently clustered together with Tr1-cells, a regulatory T-cell subset characterized by secretion of IL-10 but lacking Foxp3 expression. Gagliani et al. referred to this subset as Tr1^exTh17^. Moreover, a complete functional trans-differentiation from Th17 into regulatory Tr1-cells was suggested as these Tr1^exTh17^ cells were furthermore shown to prevent Th17-cell mediated colitis in the respective mouse model. Overall, these studies illustrate the ability of Th17-cells and Tregs to undergo phenotype conversion. The physiological role of this phenotypic and functional adaptation of these cells to changing environmental conditions may lie in maintenance of an appropriate immune-regulation in response to the presence of certain microbes, cytokines and other signals from innate immune cells. As an imbalance between Th17-cells and Tregs is observed in autoimmunity, the plasticity of these cells could be exploited in order to develop more effective therapies that will recover immune tolerance while avoiding adverse effects that go along with therapies of systemic immunosuppression.

## 5. Therapeutic Approaches: Molecules Influencing the Th17/Treg Axis

In the past years, accumulating evidence highlighted a shift in the Th17/Treg equilibrium towards the pro-inflammatory Th17-program, which is assumed to contribute to the pathogenesis of chronic inflammatory disorders such as psoriasis, PsA, RA, AS, SLE, MS, IBD and CD [2,3,4].

**Figure 2:**
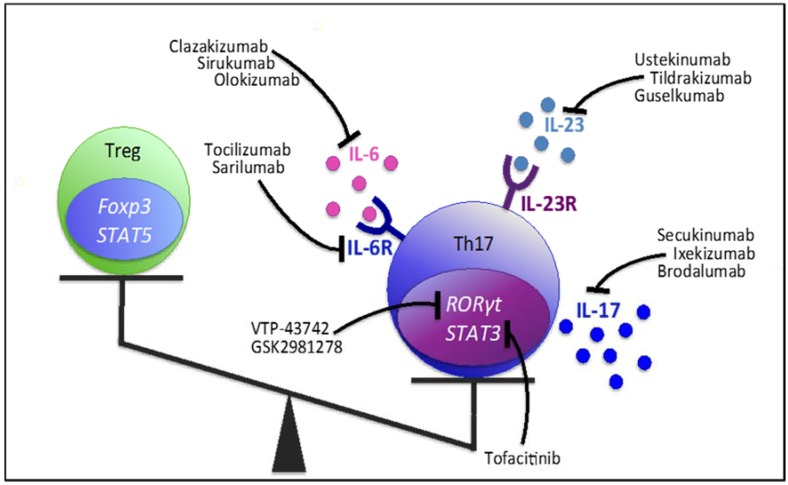
Therapeutic tools targeting Th17-cell cytokines, cytokine receptors and transcription factor pathways facilitate correction of the Th17/Treg imbalance in favor of the Treg-population. All Th17-modifying agents listed in this figure are either approved or are currently studied in clinical trials.

Various drugs with the potential to modify Treg- and in particular Th17-responses have already proven efficacy and received approval for the treatment of certain AIDs while others are currently being tested in clinical trials (Figure 2). Nevertheless, identification and testing of additional candidate molecules is an incessant research aim. Generally, therapeutic approaches are multidirectional and comprise direct targeting of Th17-related cytokines, cytokine receptors, intracellular signaling pathways, as well as inhibiting Th17- and enhancing Treg-specific transcription factors.

### 5.1. Targeting Th17-Related Cytokines and Receptors

#### 5.1.1. IL-17

IL-17 is the most prominent effector cytokine of Th17-cells and is implicated in a variety of inflammatory diseases [3]. This led to the conclusion that neutralization of IL-17, although not directly inducing a re-balance of Th17/Treg-cell ratio can abrogate IL-17-mediated pathogenic effects in autoimmune settings. Several IL-17 neutralizing monoclonal antibodies have been developed in recent years including secukinumab (AIN457), ixekizumab (LY2439821) and brodalumab (AMG827). Multiple clinical trials yielded impressive therapeutic effects of all three molecules [5,6,7,8,72] leading to the approval of secukinumab for psoriasis, PsA and AS, and ixekizumab for psoriasis [73,74,75,76]. Besides, ongoing clinical trials are evaluating the efficacy of ixekizumab in patients with active PsA (NCT01695239) and AS (NCT02696785, NCT02696798, NCT02757352).

In RA patients, IL-17 neutralization yielded inconsistent results, with some studies reporting significant clinical response and improvements in patient-reported outcomes [77,78,79], whereas others failed to meet the primary efficacy end points [80,81,82]. Importantly, approaches of IL-17 neutralization in CD were terminated due to high rates of serious adverse events and fungal infections, overall resulting in a worsening of the disease while having no beneficial impact [83,84]. The explanation for the clear failure of IL-17 neutralization in CD is most likely because of the protective role of IL-17 at mucosal surfaces where it confers host defense and contributes to the maintenance of immune homeostasis [85].

#### 5.1.2. IL-23

Years of research compellingly illustrated the requirement of IL-23 for expansion and maintenance of Th17-cells and recently also for their conversion of non-pathogenic cells with a protective role at mucosal barriers into highly pathogenic cells that promote inflammation [86,87]. IL-23 is a heterodimeric cytokine composed of two subunits, p19 and p40. The latter subunit is shared by the Th1-inducing cytokine IL-12 [88]. In psoriasis patients, p40 and p19 mRNA levels were higher in affected compared to normal skin whereas mRNA of the second IL-12 subunit (p35) was decreased in the lesions [89]. Pre-clinical psoriasis models further indicated that IL-23, but not IL-12, is able to drive excessive growth and abnormal differentiation of keratinocytes in murine skin [89]. Moreover, genetic abrogation of the p19 subunit of IL-23 in mice resulted in resistance to the development of experimental autoimmune encephalomyelitis (EAE), the most commonly used experimental model for human MS [90]. Similarly, resistance against joint inflammation was observed in p19-lacking mice with collagen induced arthritis (CIA), a mouse model of RA [91]. In this study, IL-23-deficiency was linked to the absence of IL-17-producing CD4+ T-cells [91]. Another mouse study reported that both, anti-IL-12/23p40 and anti-IL-23p19 antibodies markedly lowered transcript levels of Th17-cytokines such as IL-17 and IL-22 [92]. Blocking IL-23 and its cognate receptor IL-23R is therefore another promising therapeutic strategy for the inhibition of Th17-responses in autoimmunity.

Several antibodies for the application in humans have been developed so far, including the anti-IL-12/23p40 antibodies ustekinumab and briakinumab.

Due to its favorable efficacy, ustekinumab has already been approved for the treatment of psoriasis [93,94], PsA [95,96,97,98] and CD (NCT01369329, NCT01369342). Additional clinical trials are underway assessing the effectiveness of ustekinumab in UC (NCT02407236; phase 3), SpA (NCT02437162, NCT02438787, NCT02407223; phase 3), RA (NCT01645280; phase 2) and type 1 diabetes (T1D) (NCT02117765; phase 1 & 2). Notably, ustekinumab did not reduce disease activity of MS patients, hence no further studies are planned in this area [99]. Briakinumab was associated with an increased risk of major adverse cardiovascular events [100], and its application for marketing approval was therefore withdrawn in 2011.

Several anti-IL-23p19 antibodies (tildrakizumab, guselkumab, BI-655066, AMG 139, LY3074828) have been in clinical development. These antibodies enable specific targeting of IL-23 without the cross-reactive effect on IL-12. Tildrakizumab and guselkumab are currently under investigation in phase 3 clinical trials (Tildra: NCT01722331, NCT01729754; Guselku: VOYAGE 1 & 2) for psoriasis and in a phase 2 study for PsA (Guselku: NCT02319759). BI-655066 induced rapid and durable clinical improvements in psoriasis patients [101] while LY3074828 and AMG139 are still in early stages of studies for psoriasis, CD and UC.

#### 5.1.3. IL-6 & IL-6R

A substantial role for IL-6 in modulating the Th17/Treg ratio has become increasingly evident because IL-6 is essential for Th17-cell differentiation and also inhibits TGF-β-induced Treg-development [46,49].

Tocilizumab and sarilumab are monoclonal antibodies targeting the human IL-6R. In RA, clinical studies have demonstrated a significant reduction in Th17-cells in tocilizumab-treated patients compared to pre-treatment levels, while at the same time an increase in Tregs was seen [102,103,104]. Similarly, Tada et al. [105] reported the Foxp3/RORγt ratio to rise upon tocilizumab treatment in RA patients. An earlier study by Pesce et al. did not observe a significant effect of tocilizumab on Th17 frequencies, whereas Treg numbers increased [106]. Cytokine levels of IL-17A were not affected in tocilizumab-treated RA patients in a recent study by Lee et al. [107]; however, higher baseline IL-17A levels were associated with a worse responsiveness to the treatment. A tempting approach to achieve better treatment outcomes might thus be a combined therapy with IL-17A blockers (e.g., secukinumab) and IL-6-inhibitors such as tocilizumab. This approach, however, has yet to be addressed.

Tocilizumab is meanwhile not only approved for the treatment of RA, but also for polyarticular and systemic JIA due to its favorable results in numerous clinical trials [10,11]. In addition, sarilumab was demonstrated to significantly improve various disease-aspects in RA patients [108,109,110] but its potential approval for RA is still pending. Notably, sarilumab [111] and tocilizumab (NCT01209702, NCT01209689) both failed to achieve significant benefits in AS patients.

Sirukumab, olokizumab and clazakizumab are therapeutic tools that also interfere with IL-6 signaling, but directly target IL-6 and inhibit its interaction with the IL-6R. Several phase 3 clinical trials are currently assessing sirukumab (NCT02019472, NCT01604343, NCT01606761, NCT01856309, NCT02019472) and olokizumab (NCT02760368, NCT02760433 and NCT02760407) as therapeutic alternatives in RA. Clazakizumab already proved efficacy in phase 2 trials of RA [112] and PsA [113] and it may be expected that this drug becomes approved for the treatment of both diseases if its efficacy can be confirmed in phase 3 trials.

### 5.2. Targeting Transcription Factors

#### 5.2.1. RORγt

The ROR family of nuclear hormone receptors generally comprises three different members: RORα, RORβ and RORγ. While RORγ mRNA is present in various peripheral tissues, expression of its isoform RORγt is restricted to lymphoid tissues and certain types of lymphoid cells [114]. After RORγt was recognized as master transcription factor of the Th17-lineage, there has been a major research interest in potential inhibitors of the RORγt/Th17 axis.

The small molecule digoxin has been well established for decades in cardiology. Later it was reported also to inhibit the transcriptional activity of RORγt [115]. Digoxin suppressed differentiation of murine Th17-cells without affecting other T-cell lineages [116] and inhibited IL-17 production of T-cells from EAE mice [115]. Consistently, digoxin was effective in attenuating EAE [116] as well as in reducing the incidence of arthritis and joint inflammation in CIA mice, where it significantly reduced Th17-cells and increased Treg numbers [117].

Another study in the CIA mouse model identified the herbal medicine compound ursolic acid (UA) to effectively inhibit RORγt function and to decrease IL-17 expression in developing and differentiated Th17-cells [118]. In the spleens of these animals, UA decreased the frequency of Th17-cells while Treg numbers were increased [118]. Clinically UA treatment resulted in a reduction of disease activity of CIA mice [118].

TMP778 and TMP920 were identified as inverse agonists of RORγt [119]. Both molecules were shown to potently suppress Th17-cell generation and IL-17 secretion by differentiated Th17-cells in vitro [119]. TMP778 moreover inhibited human Th17 signature gene expression in vitro as well as murine Th17-cell differentiation in vivo [120,121]. Two independent murine studies reported TMP778 administration to reduce imiquimod-induced cutaneous inflammation (a murine model of psoriasis) and the severity of EAE [119,121]. Unfortunately, high doses of digoxin, TMP778 and TMP920 are most likely toxic for a variety of non-immune tissues and cells. Lately, Takaishi et al. [122] described attenuation of psoriasis-like lesions in two independent psoriasis mouse models after oral administration of the novel RORγt antagonist A213. They suggested that this effect was based on neutralization of IL-17-producing cells [122], however, A213 also led to a systemic attenuation of Tregs. The underlying mechanism for this effect remained unclear [122]. Smith et al. [123] described the RORγt inverse agonist GSK2981278 to significantly inhibit production of Th17 signature cytokines (IL-17A, IL-17F, IL-22 and IL-23) in multiple in vitro and human tissue-based assays, including topical delivery of the compound via the sRICA assay and psoriatic lesional explants. Assuming that tissue cytokine production is one of the main drivers of inflammation in plaque psoriasis, this study suggests that topical treatment with GSK2981278 might improve clinical outcomes of psoriasis patients [123]. Results from a recent phase 1 proof-of-concept study (NCT02548052) will shed more light on the potential benefit of topical GSK2981278 application in psoriasis patients. Another inverse agonist, VTP-43742 [124], is already in a phase II clinical trial (NCT02555709).

Given that RORγt and RORγ share a similar ligand binding domain (LBD) but differ by a variation in their N-terminal region [114], current small molecule antagonists and inverse agonist targeting RORγt bear the risk of an inadvertent impact on non-immune tissues via binding of RORγ. The mechanisms of post-translational modification of RORγt, including acetylation and ubiquitinylation, are therefore subject of ongoing investigations with the goal to develop therapeutic RORγt modulators with greater specificity, selectivity and safety.

The fact that acetylation and ubiquitination processes often compete for the same lysine residues led to speculations about the exact consequences of these modifications in Th17-cells. One hypothesis is that acetylation prevents the respective protein from ubiquitination-induced proteasomal degradation. For instance, histone acetyltransferase (HAT) p300 was shown to stabilize RORγt via acetylation of its K81 residue. Knockdown of p300 in HEK293 T-cells resulted in down-regulation of RORγt protein levels [125]. However, there is also evidence that this acetylation occurs within the DNA-binding region of RORγt consequently impairing interaction of the transcription factor with its target genes [126]. Using mass spectrometry, Lim et al. [126] reported that all three acetylated lysine residues (K69, K81, K99) located within the DNA-binding domain of RORγt become deacetylated in presence of the protein deacetylase SIRT1. Hence, SIRT1 increased transcriptional activity of RORγt and enhanced Th17-cell development and function [126]. SIRT-inhibition in EAE mice not only delayed disease onset, but also ameliorated disease severity. This observation supports the concept of a pro-inflammatory role of SIRT1 in autoimmunity [126].

Apart from acetylation, the complex area of ubiqitination-mediated RORγt-regulation was recently addressed. Several reports identified a number of E3 ubiquitin ligases (e.g., Itch, UBR5 and TRAF5) that seem to be involved in Th17-regulation by ubiquitination of RORγt [127,128,129]. E3 ligases are known to mediate the last step in the ubiquitination cascade, whereas deubiquitnating enzymes (e.g., USP15, USP17, DUBA) counteract this mechanism. Itch was identified to target RORγt for ubiquitination, resulting in decreased IL-17 expression and preventing colonic inflammation [129]. He et al. [130] recently reported that deubiquitination of K446 by ubiquitin-specific protease USP15 enhanced the recruitment of steroid receptor coactivator 1 (SRC1), thereby stimulating RORγt activity. Moreover, USP17 seems to acts as a positive regulator of RORγt [131]. Its knockdown in Th17-cells decreased RORγt protein levels and expression of Th17-related genes [131]. Paradoxically, TRAF5 mediated ubiquitination stabilizes RORγt protein levels and might thus aggravate inflammatory responses [127]. Consistent with this observation, elevated TRAF5 mRNA levels were found in CD4+ T-cells from SLE-patients [127]. Another surprising observation is the stabilization of ubiquitin ligase UBR5 upon accumulation of deubiqitinase DUBA in activated T-cells. In response to TGF-β singaling, UBR5 could then ubiquitinate the RORγ protein and act as cell-intrinsic suppressor of IL-17 production [128].

In summary, post-translational modification of RORγt is a tightly regulated, highly complex mechanism. Extensive investigational effort is needed in order to identify a safe target for novel pharmacologic interventions in AIDs.

#### 5.2.2. STAT3

Differentiation of Th17-cells is triggered by an intracellular signaling cascade involving IL-6-dependent phosphorylation and activation of STAT3 [67,132]. More precisely, binding of IL-6 to its receptor IL-6R results in homodimerization of the signal-transducing β-receptor gp130 [133]. This leads to STAT3 phosphorylation via activation of gp130-associated janus kinases (JAKs), in particular Jak1 [132]. STAT3 is a latent transcription factor that upon phosphorylation translocates into the nucleus to induce transcription of IL-6-responsive genes [132]. As previously mentioned, IL-6-dependent STAT3 activation downregulates TGF-β-induced Foxp3-expression and promotes differentiation of naïve CD4+ T-cells into Th17-cells with simultaneous suppression of Treg development [67]. Therapeutic approaches aimed at the modulation of IL-6 signaling are thus not limited to direct targeting of IL-6 and its receptor IL-6R, but also comprise inhibition of the JAK/STAT pathway.

Tofacitinib is a targeted synthetic small molecule that competitively binds to the ATP-binding pocket of JAKs, thus inhibiting their catalytic activity in a reversible manner [134]. Tofacitinib potently inhibited IL-6 induced phosphorylation of STAT1 and STAT3 in human whole blood cellular studies [135] and in synovial tissue extracts from RA patients [136]. Importantly, reduced levels of pSTAT1 and pSTAT3 in synovial biopsies were highly correlated with clinical improvement of RA, indicating that JAK1-mediated signaling of IFNs and IL-6 is involved in the synovial response to JAK blockade [136]. Moreover, tofacitinib potently suppressed the generation of pathogenic Th17-cells with an IL-23/STAT3 signature by abrogating IL-21, IL-22 but also IL-23R expression [137]. Tofacitinib is approved for RA in numerous countries [9], and it is under investigation as a possible treatment option for other inflammatory disorders , including psoriasis [138,139,140], PsA [141] and IBD [142,143]. The STAT3 inhibitor STA-21 is another compound that interferes with STAT3 signaling leading to an improvement of the clinical course of arthritis in IL-1Ra–KO mice. STA-21 enhanced Treg function and numbers while acting suppressive on Th17-cells and osteoclast formation [144]. SHR0302—another JAK inhibitor—binding JAK1 with strong affinity—reduced Th17 function along with total B-cell numbers via inhibition of JAK1-STAT3 phosphorylation in a study with adjuvant-induced arthritis rats [145]. Human clinical trials for SHR0302 have recently been started with three studies evaluating pharmacokinetics, safety and tolerability in healthy individuals (NCT02892370, NCT02423538) and RA patients (NCT02665910).

Several other biological and synthetic compounds were shown in animal models to skew the Th17/Treg balance towards the regulatory direction by blocking STAT3 phosphorylation. These compounds include the green tea-derived component epigallocatechin-3-gallate (EGCG) [146], the herbal compound celastrol [147], grape seed proanthocyanidin extract (GSPE) [148], the gastrointestinal protective drug rebamipide [149], halofuginone [150] and the antidiabetic drug metformin [151]. Among these, GSPE and metformin were also reported to increase the phosphorylation of STAT5 thus additionally enhancing Treg development [148,151].

#### 5.2.3. Foxp3

Targeting the bona fide Treg marker Foxp3 is a reasonable choice to influence the Th17/Treg equilibrium and thus, numerous studies investigated the effect of Foxp3 modulation. Several animal studies for example demonstrated that ectopic expression of Foxp3 in conventional T-cells by retroviral gene transfer resulted in a Treg-like phenotype and function and that these Foxp3-transduced T-cells were able to suppress arthritis in a murine host [152,153,154].

In the absence of IL-6, TGF-β stimulates a transcriptional program in naive T-cells with up-regulation of Foxp3 and inhibition of nuclear receptor RORγt, finally resulting in the evolvement of iTregs. Inhibition of RORγt by Foxp3, however, is abolished under the influence of TGF-β plus IL-6, leading to the generation of Th17-cells [155]. Consequently, tocilizumab treatment was shown to increase the Foxp3/RORγt ratio in patients with RA [105]. A similar effect was observed in studies evaluating the effect of TNF-α blockade on Foxp3. Etanercept treatment induced transcriptional levels of Foxp3, STAT3 and STAT4 mRNA in responding psoriasis patients [156] and increased the Foxp3/RORγt ratio in RA patients [157]. In the latter study, the Treg/Th17 ratio negatively correlated with TGF-β, but positively correlated with IL-6. More recently, adalimumab was reported to expand functional Foxp3+ Tregs via binding monocyte membrane TNF, and it unexpectedly enhanced the expression of TNF-RII on Treg-cells [158].

Abatacept, a CTLA-4 fusion protein, is clinically effective in RA and was recently effective in delaying the decline of beta-cell function in recent-onset T1D [159,160]. Tregs stimulated via CTLA-4 showed increased suppressive capacity in vitro, whereas activity of non-regulatory T-cells was reduced upon CTLA-4 ligation [161]. Moreover, abatacept therapy decreased the Foxp3/RORγt ratio [105]. In accordance with this finding, Bonelli et al. [162] observed an increase in Foxp3+ Tregs following initiation of CTLA-4Ig treatment. Tregs isolated from these abatacept-treated patients, however, revealed diminished in vitro suppression of responder cell proliferation [162].

In the early phase of B-cell depletion with rituximab, mRNA levels of Foxp3 were significantly increased in patients with active SLE. During follow-up, patients in clinical remission revealed persistently elevated Foxp3 mRNA levels, whereas this marker decreased in patients with active disease [163]. This finding is potentially explained by a simultaneous rise in mRNA levels of TGF-β, a cytokine contributing to Treg induction.

Apart from the aforementioned factors, epigenetic modifications contribute to the regulation of Foxp3 expression. A critical factor in Foxp3 protein stability is the methylation status of CpG sites within the proximal promoter region of the *FOXP3* gene [164]. In vitro experiments showed that TGF-β favors de-methylation, whereas the addition of IL-6 increases methylation, the latter resulting in reduced Foxp3 expression [165]. More recently, Cribbs et al. [166] reported that methotrexate was able to increase expression of Foxp3 and restored suppressive function of initially defective Tregs in RA patients. Bisulfite sequencing PCR of methotrexate-treated Tregs revealed a significant reduction in methylation of the *FOXP3* upstream enhancer region [166]. Similarly, treatment with the combination rapamycin/IL-2 was shown to augment the frequency and function of Tregs in vitro [167]. The suppressive activity of these Tregs was further increased by all trans retinoic acid, leading to a lower methylation status of the *FOXP3* gene [168].

These findings led to the hypothesis that inhibition of DNA methyltransferases (DNMTs) might be a treatment option in autoimmunity. In vitro studies demonstrated the emergence of a stable Foxp3+ Treg population in the presence of TGF-β and the DNMT inhibitor 5-aza-20- deoxycytidine [165]. Another method to increase Foxp3 expression in Tregs is the inhibition of histone deacetylases (HDACs). HDACs are normally recruited by methylated DNA and mediate compact nucleosome formation. A study in mice showed that trichostatin A therapy down-regulating HDACs led to increased prevalence and suppressive function of Foxp3+ Tregs [169]. Accordingly, impaired Treg function was observed upon inhibition of acetyltransferase p300, since p300—despite increasing stability of RORγt [125]— hyperacetylates Foxp3 [170]. Besides, deletion of HDAC6, HDAC9, or Sirt1 increased expression of the gene encoding Foxp3, and enhanced suppressive function of Tregs [171,172,173]. On the other hand, Tregs of HDAC5^−/−^ mice showed reduced suppressive function in vitro and in vivo. CD4+ T-cells lacking HDAC5 convert poorly into Tregs despite appropriate polarizing conditions [174]. Therefore, there is a need for subsequent studies and a more sophisticated inhibition of HDACs seems necessary to ensure stable Treg induction.

#### 5.2.4. FoxO1

Recently, FoxO1 was reported to be a negative regulator of the Th17 transcriptional program [175]. Expression of FoxO1 in T-cells resulted in a distinct reduction in Th17 generation as well as in a lower transcription of IL-17 and IL-23R genes in vitro. At the molecular level, FoxO1 binds RORγt via its DNA binding domain thereby inhibiting the activity of this gene. As outlined above, RA patients treated with the anti-IL-6R antibody tocilizumab yielded a marked reduction of circulating Th17-cells and simultaneously an increase of peripheral Tregs [102]. The effect of IL-6 is related, at least partly, to FoxO1. Ichiyama et al. [176] showed that IL-6 induces a microRNA-183-96-182 cluster in Th17-cells. This cluster directly represses FoxO1 which in turn inhibits the expression of IL-1R1 resulting in enhanced pathogenic cytokine production [176]. Another study reported that microRNA 182 inhibits Treg differentiation in a FoxO1 dependent manner [177]. Accumulating evidence suggests that anti-TNF-α therapy may support immunomodulation by Tregs via the influence of this therapy on FoxO1. While TNF-α induced the activation of FoxO1 in human fibroblasts [178], the TNF-α antagonist etanercept was reported to re-activate FoxO1 in patients suffering from psoriasis [179]. Liao et al. [180] reported that TNF-α promotes microRNA-705 expression, which in turn represses FoxO1 via post-transcriptional regulation. Consistently, elevated numbers of Tregs [181] as well as reduced frequencies of Th17-cells [182,183] were described following anti-TNF-α treatment in patients with RA.

FoxO1 is also modulated by environmental factors. Wu et al. reported for example that a modest increase in salt concentration induces SGK1 expression thus deactivating FoxO1 and promoting IL-23R expression and Th17-cell differentiation in mice in vitro and in vivo [65]. Besides, in vitro high-salt activation of human Tregs resulted in the loss of suppressive function that was mediated by FoxO1-dependent alterations in Foxp3 stability [184]. Luo et al. recently reported that differentiation of activated Tregs was associated with repression of FoxO1-dependent gene transcription, along with a reduced FoxO1 expression [185]. This finding indicates that the role of FoxO1 is not fully understood and requires further investigation.

### 5.3. Targeting Intracellular Signaling Pathways

#### 5.3.1. ROCK Inhibition

Rho associated kinase 2 (ROCK2) was shown to be activated in murine T-cells under Th17- conditions and is thought to influence Th17-generation via phosphorylation of IRF4, a transcription factor involved in the development of Th17-cells [186,187]. Notably, ROCK activity was found to be increased in SLE and RA patients [188,189]. In studies on human T-cells, ROCK2 was reported to control IL-21 and IL-17 secretion via mechanisms that involve regulation through STAT3, IRF4, and RORγt [190,191]. As reported by Biswas et al. [187] ROCK inhibition diminished production of IL-17 and IL-21 and ameliorated disease signs in Def6^trap/trap^DO11.10 mice, a murine model of RA-like arthritis. Fasudil derivative FaD-1, a ROCK inhibitor, was reported to ameliorate neurological defects and disease severity in EAE mice, in part via downregulation of the Th17-response in the spinal cord [192]. WAR-5, another ROCK inhibitor described to selectively inhibit ROCK2 led to the attenuation of myelin damage, reduction of CNS inflammation and alleviation of clinical symptoms in EAE mice [193]. WAR-5 treatment further reduced levels of IFN-γ and IL-17 while increasing IL-10. This might thus skew the imbalance between Th1/Th17-cells and Tregs in many autoimmune diseases toward immune regulation [193]. Zanin-Zhorov et al. [190] investigated KD025, a selective ROCK2 inhibitor, to modulate inflammation by decreasing STAT3 activation, enhancing STAT5 phosphorylation and increasing the suppressive function of Tregs. Overall, KD025 induced a beneficial shift in the Th17/Treg balance [190]. Two phase 2 clinical trials (NCT02317627 and NCT02106195) investigating the efficacy of KD025 for the treatment of psoriasis have recently been completed, the results of these trials will be available soon. Another study with a similar objective (NCT02852967) has just started recruitment and will presumably be completed in 2018.

#### 5.3.2. MAPK Inhibition

In contrast to effector T-cells, Tregs constitutively express GITR at a high level [23]. Interestingly, ligation of GITR with its cognate ligand GITRL can abrogate suppressive function of Tregs [194]. The differentiation of naïve CD4+ T-cells was skewed toward the Th17-lineage when cultured with GITRL protein. Besides, the administration of recombinant GITRL resulted in enhanced Th17-cell generation and exacerbation of arthritis in CIA mice [195].

Tang et al. [196] have proposed the molecular mechanism underlying GITRL modulation of Th17-cells to be based on enhanced phosphorylation of p38 MAPK and subsequent phosphorylation of STAT3. Elevated levels of p38 MAPK phosphorylation in RA CD4+ T-cells correlated with increased serum autoantibody levels in these patients [196]. More recently, Li et al. [197] reported that elevated concentrations of GITRL were found in RA synovial fluid and serum, and that GITRL levels correlated with autoantibody production. Notably, administration of a p38 MAPK inhibitor resulted in suppression of GITRL-induced Th17-differentiation and ameliorated disease activity in CIA mice [196]. Unfortunately, several specific pharmacologic inhibitors of p38 MAPK inhibitors were found to be ineffective in clinical studies of RA [198].

## 6. Conclusions

A bulk of evidence has highlighted a shift of the Th17/Treg equilibrium towards the pro-inflammatory Th17-program in several autoimmune disorders, contributing to their progression and recurrent clinical exacerbation. Substantial progress in understanding the development, function and reciprocal regulation of Th17 cells and Tregs yielded a high therapeutic potential for targeting these cell populations. The pathophysiology of autoimmunity however, is complex, explaining the observations that certain therapeutic strategies are effective in some AIDs (e.g., IL-6R blockade in RA [10]), while exhibiting no benefit in others (e.g., IL-6R blockade in AS [111]). Attention must furthermore be drawn to the physiologic role of Th17-cells and -cytokines. For example, IL-17 confers host defense and, in addition to other Th17-cytokines like IL-22, participates in the maintenance of immune homeostasis at mucosal surfaces, especially in the gut [85]. IL-17 neutralization was accompanied by high rates of serious adverse events and fungal infections in CD patients [83,84], despite being highly effective in the treatment of psoriasis [5,6,7,8,72]. Recently, it was shown that Th17 is not a uniform subset but rather consists of a non-pathogenic and a pathogenic cell population [61,62,63,64,65,66]. Targeting pathogenic Th17-cells alone might therefore be the next step to ameliorate Th17/Treg-targeted therapies. Moreover, little is known about the consequences of long-term inhibition of the IL-17 pathway. Various novel candidate molecules to beneficially re-shape the Th17/Treg imbalance have recently shown promising results in animal models [116,118,120,122]. The application of these substances still poses safety issues in humans and requires further evaluation before it can be tested in clinical trials in humans.

In summary, there are still multiple challenges to identify, develop and implement the “ideal” Th17/Treg-targeted interventional strategy with respect to the treatment of autoimmunity.
